# Supplementary material for: Screening of Differentially Expressed Microsporidia Genes from Nosema ceranae Infected Honey Bees by Suppression Subtractive Hybridization
Source: Insects. 2020 Mar 22;11(3):199. doi: 10.3390/insects11030199 (PMC7143254; doi:10.3390/insects11030199)
Supplement: Supplementary file 1 [file insects-11-00199-s001.pdf]

Table S1. Primer sets list for this study.

No.	Gene	Primer Name	Sequence (5' to 3')	Tm (℃)	Note
1	SSU rDNA	18f	CACCAGGTTGATTCTGCC	50	Huang et al., 2004 [42]
		1537r	TTATGATCCTGCTAATGGTTC		
2	18S	143F	TGCCTTATCAGCTNTCGATTGTAG	50	Lo et al., 1997 [43]
		145R	TTCAGNTTTGCAACCATACTT CCC		
3	NCER_100435	F	GCACACCTGGATCAACAGAGAA	59	This study
		R	CTTAAGAACAAGTGCACCAGATCTA		
4	NCER_101664	F	GCTCTTGTTGTTGACAATGGTTC	59	This study
		R	AGCATCATCCCCAGCAAATC		
5	NCER_100566	F	TTGATTCTAGCCACTTTTACCGA	59	This study
		R	ACCCGAGAAGCTGCAGATATTAA		
6	NCER_100064	F	CGTGCAAATTAAAAGAAGATATTATTAA	59	This study
		R	CCATCATACCTGCAAAAGTCATAG		
7	NCER_101665	F	CAATACACGCAAAGAAAACCAAAT	60	This study
		R	TCCTTCAGCGGATTCTAAAGC		
8	NCER_101348	F	ATGGAAAACGATGAATACGCTG	60	This study
		R	GCCATGTCTTCATATCTTTCTGCTA		
9	NCER_100249	F	GTATACCACCAGATCAACAGAGACTT	59	This study
		R	TCATTAAGTGTTCTTCCATCTTCCA		
10	NCER_101591	F	CTACAATACAATCACTTCCAGGTCTT	59	This study
		R	GTGCGACTCCCAATAAACCAT		
11	NCER_101194	F	TGGATGAACTTTGTTGTTGGTGT	59	This study
		R	TTATGTCCTTGGATTAGTTCTCTTGA		
12	18S-qPCR	18S rRNA-F	GTAACCCGTTGAACCCCATT	59	Schmittgen et al., 2000 [44]
		18S rRNA-R	CCATCCAATCGGTAGTAGCG		

Table S2. Gene ontology analysis of forward library and reverse library.

GO Classification	GO term	No. of EST	
		Forward library	Reverse library
Molecular Function	catalytic activity (GO:0003824)	5	4
	binding (GO:0005488)	3	4
	transporter activity (GO:0005215)	3	3
	molecular function regulator (GO:0098772)	1	1
	structural molecule activity (GO:0005198)	-	1
	transcription regulator activity (GO:0140110)	-	1
Biological Process	cellular process (GO:0009987)	4	4
	response to stimulus (GO:0050896)	2	2
	cellular component organization or biogenesis (GO:0071840)	1	1
	metabolic process (GO:0008152)	-	5
	biological regulation (GO:0065007)	-	2
	localization (GO:0051179)	-	1
Cellular Component	membrane (GO:0016020)	4	4
	cell (GO:0005623)	3	5
	protein-containing complex (GO:0032991)	2	1
	organelle (GO:0043226)	1	3

References

42. Huang, W.F.; Tsai, S.J.; Lo, C.F. et al. The novel organization and complete sequence of the ribosomal RNA gene of *Nosema bombycis*. *Fungal. Genet. Biol.* **2004,** 41, 473-481. doi: 10.1016/j.fgb.2003.12.005
43. Lo, C.F.; Ho, C.H.; Chen, C.H. et al. Detection and tissue tropism of white spot syndrome baculovirus (WSBV) in captured brooders of Penaeus monodon with a special emphasis on reproductive organs. *Dis. Aquat, Org.* **1997,** 30,53-72. doi:10.3354/dao030053

44. Schmittgen, T.D.; Zakrajsek, B.A. Effect of experimental treatment on housekeeping gene expression: validation by real-time, quantitative RT-PCR. J. Biochem. Biophys. Methods 2000, 46, 69-81. doi: 10.1016/S0165-022X(00)00129-9.
